# Gray matter abnormalities in Tourette Syndrome: a meta-analysis of voxel-based morphometry studies

**DOI:** 10.1038/s41398-021-01394-8

**Published:** 2021-05-14

**Authors:** Xinyue Wan, Simin Zhang, Weina Wang, Xiaorui Su, Jun Li, Xibiao Yang, Qiaoyue Tan, Qiang Yue, Qiyong Gong

**Affiliations:** 1 Huaxi MR Research Center (HMRRC), Department of Radiology, West China Hospital of Sichuan University, Chengdu, 610041 China; 2 Department of Radiology, The First Affiliated Hospital, College of Medicine, Zhejiang University, Hangzhou, 310000 China; 3 The Center of Gerontology and Geriatrics, West China Hospital of Sichuan University, Chengdu, 610041 China; 4 Department of Radiology, West China Hospital of Sichuan University, Chengdu, 610041 China; 5 Research Unit of Psychoradiology, Chinese Academy of Medical Sciences, Chengdu, 610041 China; 6 Functional and Molecular Imaging Key Laboratory of Sichuan Province, Chengdu, 610041 China

**Keywords:** Neuroscience, Diseases

## Abstract

Tourette syndrome (TS) is a neurobehavioral disorder for which the neurological mechanism has not been elucidated. Voxel-based morphometry (VBM) studies have revealed abnormalities in gray matter volume (GMV) in patients with TS; however, consistent results have not been obtained. The current study attempted to provide a voxel wise meta-analysis of gray matter changes using seed-based d mapping (SDM). We identified ten relevant studies that investigated gray matter alterations in TS patients and performed a meta-analysis using the SDM method to quantitatively estimate regional gray matter abnormalities. Next, we examined the relationships between GMV abnormalities and demographic and clinical characteristics. Our results demonstrated that TS patients had smaller GMV in the bilateral inferior frontal gyri and greater GMV in the cerebellum, right striatum (putamen), and bilateral thalami (pulvinar nucleus) than healthy controls. A meta-regression analysis did not identify correlations between GMV changes and demographic or clinical variables. This meta-analysis confirmed significant and consistent GMV changes in several brain regions of TS patients, primarily in the cortico-striato-thalamo-cortical network.

## Introduction

Tourette syndrome (TS) is a neurological disorder characterized by primary motor and vocal tics, and it is frequently concomitant with obsessive–compulsive disorder (OCD), attention-deficit–hyperactivity disorder (ADHD), or other social and behavioral disturbances^1–3^. TS is classically identified in 5 individuals per 1000^4^, tends to be inherited through families and often affects boys^5^ with a peak onset age of 3–8 years^6^. Previous studies have suggested that the severity of tics and comorbidities are age-related and may gradually achieve remission during adolescence^7,8^, and TS patients with comorbid disorders are at higher risk of suicide than pure TS patients^9^. The clinical symptoms of TS are complex and difficult to treat and thus pose major public health and economic burden. To date, the neurological basis of TS has not been determined.

In the past decade, neuroimaging technologies have been applied to studies of TS, among which voxel-based morphometry (VBM) is one of the most widely used magnetic resonance imaging (MRI) technologies. VBM is a comprehensive analysis technology for brain structure that can reflect the anatomical changes in the brain through quantitative calculation and analysis^10,11^. Although a previous VBM study^12^ did not find any brain morphological difference between treatment-naive boys with pure TS and healthy controls (HCs), other studies^13–17^ did find significant changes in TS patients. However, controversial changes have been reported by different studies. Reduced GMV was found in sensorimotor areas, the left superior temporal gyrus, left caudate nucleus, left postcentral gyrus, left hippocampal gyrus, bilateral anterior cingulate cortices and frontal areas (including the left frontal pole, bilateral inferior frontal gyri (IFG) and orbitofrontal, ventrolateral prefrontal cortices)^15,17–21^. Increased GMV has also been reported; for example, Garraux et al.^14^ found greater midbrain volumes in TS patients, and another study^13^ also found increased GMV in the posterior thalamus and hypothalamus. The inconsistency may be caused by differences in sample size and demographic and clinical characteristics or the effects of imaging techniques. These inconsistencies increase the difficulty of understanding the neurological mechanism of TS, and further exploration must be performed to reach a consensus. The meta-analysis method can provide a precise and robust summary after synthesizing the multitude of results from different studies in an unbiased way, and it may offer insights that are not immediately apparent from the individual studies^22^. Therefore, we performed a meta-analysis to integrate several previous studies with inconsistent results.

We hypothesized that some functional brain regions (such as the frontal cortex^23,24^, thalamus^25,26^, hippocampus^27^, basal ganglion, and midbrain^28^) of TS patients may be affected and thus show structural changes and that GMV abnormalities might be related to certain clinical factors. Therefore, we first performed a pooled meta-analysis of all the included VBM studies to determine the most prominent and consistent changes in gray matter in TS patients. Seed-based d mapping (SDM) software was used in this process because it can control the results of individual studies and all the information included in the study can be used in the same map^29^. We also analyzed the robustness and heterogeneity of the main findings. Next, a multivariate meta-regression analysis was performed to explore the potential relationship between the GMV changes and the clinical and demographic characteristics.

## Methods

### Selection of studies

A systematic search was performed for relevant studies published in the PubMed, Web of Science, Embase, and Science Direct databases before July 31, 2020, according to the “Preferred reporting items for systematic reviews and meta-analyses” (PRISMA) guidelines^30^. The key search words were ‘Tourette syndrome’ or ‘Gilles de la Tourette syndrome’ or ‘TS’ or ‘GTS’ plus ‘voxel-based morphometry’ or ‘VBM’ or ‘voxel-based’ or ‘voxel-wise’ plus ‘magnetic resonance imaging’ or ‘MRI’. Manual searches were also conducted in the reference lists of these studies.

The inclusion criteria of the studies were as follows: (i) original research published in peer-reviewed English journals; (ii) studies using VBM to analyze the changes in GMV of the whole brain; (iii) studies comparing the GMV between TS patients and HCs; and (iv) studies reporting whole-brain results in a stereotactic space (MNI or Talairach). Studies were excluded if they (i) were meta-analyses, case reports or reviews; (ii) failed to provide the three-dimensional coordinates in stereotactic space; or (iii) failed to include HC controls. If several publications were based on the same study, only the paper reporting the largest sample size was selected.

**Fig. 1:**
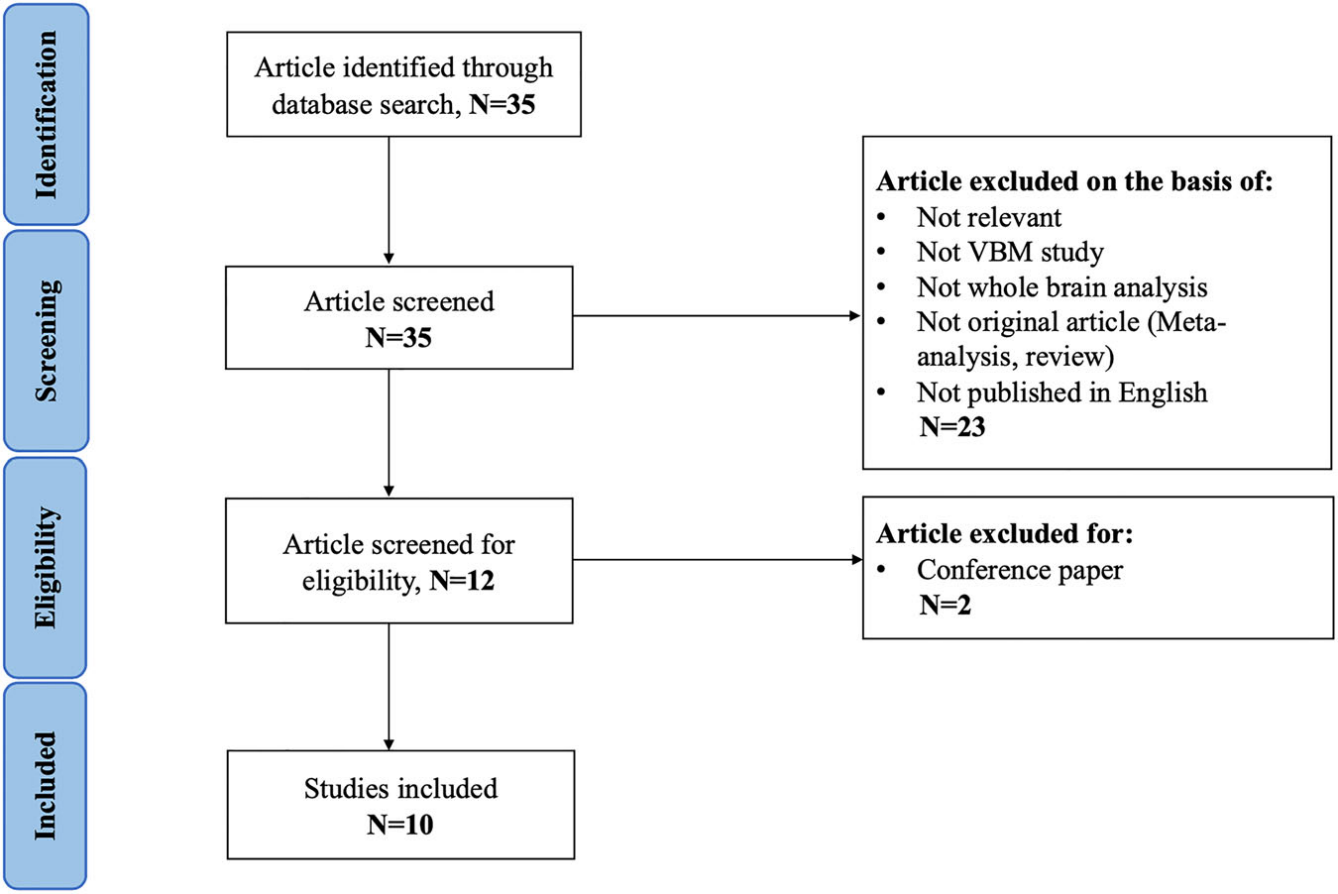
Search strategy used for the inclusion of the studies considered in the current meta-analysis. *Abbreviation*: VBM voxel-based morphometry.

Two authors (X.Y.W. and S.M.Z.) searched the literature independently, checked all articles, and extracted and cross-checked the data. In case of a difference of opinion, they discussed the findings until a consensus was reached. The research screening process is shown in Fig. 1. According to the SDM method, we extracted the following data from the included studies: demographic and clinical characteristics (sample size, age, illness duration, onset age, attention-deficit hyperactivity disorder self-assessment scale (ADHS-SR), Yale–Brown obsessive–compulsive scale (Y-BOCS)^31^, Yale Global Tic Severity Scale (YGTSS)^32^, percentage of medicated patients and comorbidity), technical details (MRI scanner, software, smoothing, *p* value, and voxels) and three-dimensional coordinates.

### Voxel-wise meta-analysis: SDM

SDM is a statistical method for meta-analyses of brain activity or structural differences identified via neuroimaging techniques. SDM retains the useful features of original research and makes some improvements on the basis of some methods, such as activation likelihood estimation (ALE) and multi-level kernel density analysis (MKDA)^33^. We intended to conduct a pooled meta-analysis of all the included studies. Before we performed the meta-analysis, the peak coordinates and corresponding *t*-values were extracted from each study, and *p* values or *z*-values were converted to *t*-values online (http://www.sdmproject.com/utilities/?show=Statistics). Using SDM software (https://www.sdmproject.com/), we used the peak coordinates to recreate a map of the effect size. We performed all analyses based on the detailed analysis workflow described in the SDM tutorial (https://www.sdmproject.com/old/) as well as some publications^34–36^. First, positive and negative coordinates were reconstructed on the same map; thus, a signed differential map was obtained. Second, using effect sizes, reported peak coordinates were combined with statistical parametric maps for more accurate meta-analyses. Third, the heterogeneity, robustness and publication bias of the results were assessed (three analyses were used in our study). In SDM v4.31, a standard Montreal Neurological Institute (MNI) map of the GMV differences was recreated for each included study using an anisotropic Gaussian kernel that assigns higher effect sizes to the voxels that are more closely correlated with peaks. These anisotropic kernels are introduced to optimize the recreation of effect size maps and provide greater robustness because they do not depend on a full width at half maximum^37^.

We assessed the robustness of the results using a jack-knife sensitivity analysis. It was performed to verify the reliability and stability of the findings through systematically repeated meta-analyses by excluding one study at a time. With the same threshold, inter-study heterogeneity analysis was conducted to find the heterogeneous brain regions with *Q* statistics using a random-effects model (*Q* maps can show the brain regions with significance inter-study heterogeneity). In addition, Egger’s test was used to assess publication bias by STATA software^38^. Meta-regression analyses were conducted using clinical variables including age, illness duration, ADHS-SR, Y-BOCS, YGTSS, and percentage of medicated patients, as regressors. In addition, we used MRIcron software (http://www.mricro.com/mricron/) to convert the final SDM results into images.

According to the recommendation of the developers of the SDM method, a threshold of *p* < 0.005 with a peak *z* > 1 and a cluster extent of more than 10 voxels were used for the meta-analyses (the default SDM thresholds)^34^. A conservative threshold of *p* < 0.0005 was adopted in the meta-regressions^29,34^.

## Results

### Included studies and sample characteristics

Based on the above strategy, a total of 35 studies were initially identified, and 10 of them met the inclusion criteria. These studies included 331 TS patients and 327 HCs. Table 1 summarizes the demographic and clinical data of all the included studies. Table 2 summarizes the technical details. Details of VBM preprocessing are summarized in Supplementary Table S1. None of the studies identified significant differences in age or sex between the TS group and the matched HC group. Supplementary Table S2 shows the results of GMV alterations from original studies while the original coordinates and respective effect sizes are reported in Supplementary Table S3.

**Table 1:** Demographic and clinical characteristics of subjects in the 10 voxel-based morphometry data sets included in the meta-analysis.

Study	Number (male)		Age (years)		Duration	ADHS-SR*	Y-BOCS	YGTSS	Medication (%)	Comorbidity
	TS	HC	TS	HC	(years)					
Garraux et al.^14^	31 (25)	31 (23)	32.00 ± 10.50	32.00 ± 11.00	NA	NA	NA	31.60	29.00	Yes
Ludolph et al.^17^	14 (14)	15 (15)	12.50	13.40	NA	NA	NA	NA	28.00	Yes
Muller-Vahl et al.^18^	19 (19)	20 (20)	30.40	31.70	NA	26.88	NA	28.80	Drug naive	No
Roessner et al.^12^	38 (38)	38 (38)	11.89 ± 1.33	12.19 ± 1.38	5.40 ± 2.00	NA	NA	NA	Drug naive	No
Draganski et al.^20^	40 (30)	40 (25)	32.40 ± 11.00	34.40 ± 9.00	24.00 ± 11.60	NA	NA	28.70 ± 7.40	62.50	Yes
Wittfoth et al.^15^	29 (29)	24 (24)	30.70 ± 9.00	30.60 ± 10.90	NA	7.20	10.50	35.70 ± 17.60	Drug free	Yes
Liu et al.^19^	21 (20)	20 (17)	7.90 ± 1.95	8.05 ± 2.30	1.84 ± 0.56	NA	NA	41.71 ± 12.46	Drug free	No
Ganos et al.^21^	14 (13)	15 (13)	30.60 ± 8.80	31.60 ± 8.90	NA	2.30 ± 2.40	0.90 ± 1.80	17.60 ± 6.60	21.40	No
Debes et al.^16^	22 (20)	21 (NA)	14.20 ± 2.50	NA	NA	NA	NA	NA	Drug free	Yes
Greene et al.^13^	103 (81)	103 (81)	11.90 ± 2.10	11.90 ± 2.10	NA	NA	5.30 ± 6.80	18.10 ± 8.30	72.82	Yes

*TS* Tourette syndrome, *HC* health control, *ADHS-SR* attention-deficit hyperactivity disorder self-assessment scale, *Y-BOCS* Yale–Brown obsessive–compulsive scale, *YGTSS* Yale Global Tic Severity Scale, *NA* not available.

**Table 2:** Technique details of the VBM studies on TS included in the meta-analysis.

Study	MRI scanner	Head coil	T1 sequence	Software	Smoothing (FWHM)	*p* Value	Voxels	Coordinates	Template	Voxels size (image acquisition)
Garraux et al.^14^	3.0 T (GE)	A standard GE head coil	FSPGR	SPM2	8 mm	*p* < 0.050 (FDR)	NA	3	MNI	1.3 × 0.97 × 0.97 mm^3^
Ludolph et al.^17^	1.5 T (Siemens)	NA	NA	SPM2	6 mm	*p* < 0.001 (corrected)	NA	4	MNI	NA
Muller-Vahl et al.^18^	1.5 T (GE)	NA	SPGR	SPM2	8 mm	*p* < 0.050 (FWE)	NA	14	MNI	0.97 × 0.97 × 1.5 mm^3^
Roessner et al.^12^	3.0 T (Siemens)	8-channel	MPRAGE	SPM5	8 mm	*p* < 0.050 (FWE)	NA	0	MNI	1 × 1 × 1 mm^3^
Draganski et al.^20^	1.5 T (Siemens)	a phased-array coil	MDEFT	SPM8	6 mm	*p* < 0.050 (FWE)	NA	8	MNI	1 × 1 × 1 mm^3^
Wittfoth et al.^15^	1.5 T (GE)	NA	SPGR	SPM8	8 mm	*p* < 0.001 (uncorrected)	NA	1	MNI	0.97 × 0.97 × 1.5 mm^3^
Liu et al.^19^	1.5 T (Philips)	NA	3D-FFE	SPM8	6 mm	*p* < 0.001 (uncorrected)	NA	3	MNI	1 × 1 × 1 mm^3^
Ganos et al.^21^	3.0 T (Siemens)	12-channel	MPRAGE	SPM8	8 mm	*p* < 0.001 (corrected)	NA	2	NA	1 × 1 × 1 mm^3^
Debes et al.^16^	3.0 T (Philips)	8-channel	Turbo-GRE	FSL	3 mm	*p* < 0.050 (corrected)	NA	1	MNI	1 × 1 × 1 mm^3^
Greene et al.^13^	3.0 T*6/1.5 T*2 (several)	several	MPRAGE	SPM12	6 mm	*p* < 0.001 (FDR)	NA	9	MNI	Several (1.0–1.25 mm^3^)

*VBM* voxel-based morphometry, *TS* Tourette syndrome, *MRI* magnetic resonance imaging, *GE* general electric, *FSPGR* fast spoiled gradient recalled sequence, *SPGR* spoiled gradient recalled echo sequence, *MPRAGE* magnetization-prepared rapid acquisition of gradient echoes sequence, *MDEFT* modified driven equilibrium Fourier transform protocol, *3D-FFE* three-dimensional-Fast Field Echo sequence, *Turbo-GRE* turbo gradient echo sequence, *FWHM* full-width at half-maximum, *SPM* statistical parametric mapping, *FDR* false discovery rate, *FWE* family-wise error correction, *NA* not available, *MNI* Montreal Neurological Institute Space.

### Pooled meta-analysis of all the included studies

**Fig. 2:**
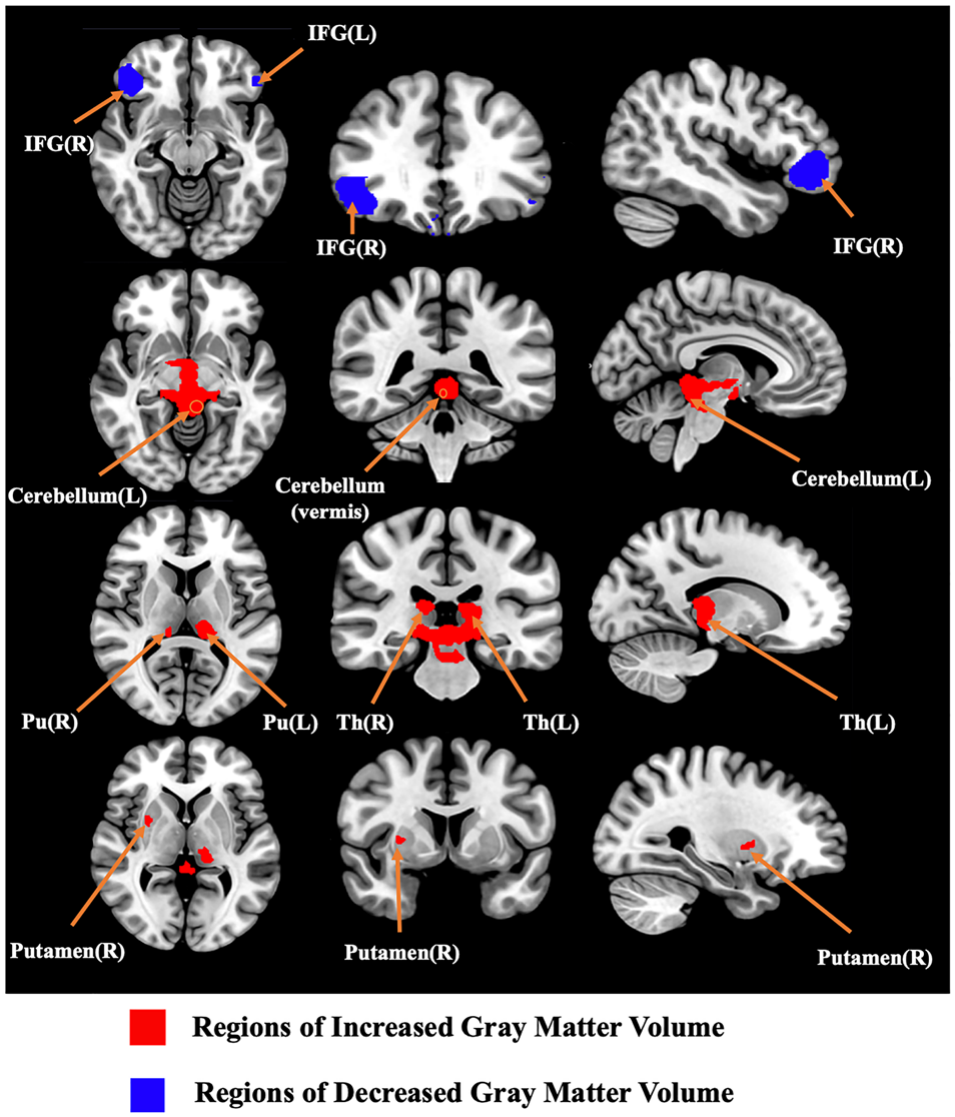
Regions showing gray matter volume alterations in TS patients compared with healthy controls. *Abbreviation:* TS Tourette syndrome, L left, R right, IFG inferior frontal gyrus, Pu the pulvinar nucleus, Th thalamus.

TS patients showed decreased GMV in the bilateral IFG. On the other hand, there were also some regions where GMV increased, i.e., the cerebellum, right striatum, and bilateral thalami (Table 3 and Fig. 2). Three-dimensional rendering images are shown in Supplementary Fig. S1.

**Table 3:** The brain regions with altered gray matter volume in TS patients identified by the main meta-analyses.

Region	Maximum				Cluster	Jackknife sensitivity analysis	Egger’s tests (*p*)
	MNI coordinates *x*, *y*, *z*	SDM *z*-score	*p* Value uncorrected	Number of voxels	Breakdown (no. of voxels)		
*Increased regions*							
Cerebellum, vermic lobule III	2, −36, −12	2.363	<0.000000001	192	Left cerebellum, hemispheric lobule III/IV/V, BA27 30 (98) Cerebellum, vermic lobule I/II/III/IV/V, BA 27 30 (91) Right cerebellum, hemispheric lobule III (3)	9 of 10	0.030
Left thalamus	−10, −28, 10	2.113	0.000051618	112	Left thalamus (96) Left thalamus, BA 27 (16)	9 of 10	0.002
Right striatum	8, −6, −12	2.192	0.000020623	72	Right striatum (52) Right lenticular nucleus, putamen (20)	10 of 10	0.005
Right thalamus	12, −28, 14	1.992	0.000129044	54	Right thalamus (48) Right thalamus, BA 27 (6)	9 of 10	0.002
*Decreased regions*							
Right inferior frontal gyrus	42, 36, −4	−1.507	0.000129044	635	Right inferior frontal gyrus, orbital part (468) Right inferior frontal gyrus, triangular part (107) Right middle frontal gyrus (60)	9 of 10	0.457
Right supramarginal gyrus	60, −14,24	−1.348	0.000696719	134	Right supramarginal gyrus, BA 42 43 48 (50) Right rolandic operculum, BA 22 42 48 (84)	8 of 10	0.477
Right postcentral gyrus	52, 12, −12	−1.192	0.000696719	129	Right postcentral gyrus, BA 3 43 48 (126) Right postcentral gyrus (3)	8 of 10	0.279
Left inferior frontal gyrus	−52, 28, −8	−1.082	0.004154444	90	Left inferior frontal gyrus, orbital part (57) Left inferior frontal gyrus, triangular part (33)	7 of 10	0.676
Left rolandic operculum, BA 48	−56, −6, 12	−1.267	0.001491487	81	Left rolandic operculum, BA 48 (79) Left rolandic operculum (2)	8 of 10	0.249
Left superior temporal gyrus	−52, −14, 12	−1.207	0.001852751	56	Left superior temporal gyrus, BA22 42 48 (56)	8 of 10	0.218

*TS* Tourette syndrome, *BA* Brodmann area, *MNI* Montreal Neurological Institute Space, *SDM* seed-based d mapping, *BA* Brodmann area.

### Reliability analysis

When the whole-brain jackknife sensitivity analysis was performed, the main findings remained largely unchanged. The detailed data are shown in Table 3. We performed a visual inspection of the heterogeneity analysis according to the AES-SDM tutorial. It revealed that no significant inter-study heterogeneity occurred in the regions reported in the results (*p* > 0.005). The published bias results are summarized in Table 3.

### Meta-regression analysis

We performed a simple linear regression analysis using SDM software to examine the potential impact of relevant clinical variables on GMV changes. Mean age, YGTSS, and percentage of medicated patients showed no relationship with the GMV changes. Due to the limited datasets included in this study, we failed to conduct the meta-regression analysis for illness duration, ADHS-SR, and Y-BOCS.

## Discussion

Our study pooled 10 VBM studies with 331 TS patients and 327 controls to define GMV differences between TS patients and HCs. We observed the following gray matter alterations: (1) TS patients showed decreased GMV in the bilateral IFG; (2) TS patients showed increased GMV in the cerebellum, right striatum, and bilateral thalami; and (3) clinical or demographic characteristics were not correlated with GMV alterations based on the meta-regression analysis. To the best of our knowledge, this is the first meta-analysis performed to investigate whether consistent GMV alterations occur in TS patients using SDM.

Previous research methods, such as the region-of-interest approach, often focus on selected regions and exclude exploration of other brain regions that may be involved. Based on VBM, we can detect effects that do not fit traditional anatomic boundaries, such as a blob that is mostly located in the right IFG and partly in the right middle frontal cortex (Table 3). SDM is a widely applied method for neuropsychiatric studies^39–42^. Compared with ALE and MKDA, SDM is an optimal voxel-based meta-analytic method that adopts and combines the positive features of these two methods^29,34^. A novelty of this method is that both positive and negative coordinates are reconstructed in the same map to obtain a signed differential map, which represents an important feature to prevent the occurrence of both positive (increased volume or activation) and negative (decreased volume or activation) results of a particular voxel^29^. SDM can analyze the robustness of the results, which will ensure that the final results are the most replicable and robust. Furthermore, this method is capable of weighting and controlling results for multiple moderators including demographics, clinical information, and imaging factors. Another function of SDM is to conduct meta-analysis group comparisons to detect whether the computed effect sizes differ significantly between subgroups^37^. However, the number of included studies in our study was limited and could not meet the minimum requirement for subgroup analyses (ten studies)^42^. Some brain regions in the results may have potential publication bias and need verification in future studies. First, we performed a comprehensive literature search. Second, there is a tendency to publish studies with positive rather than negative results. In fact, when the number of studies is small (less than 20), the sensitivity of Egger’s tests for publication bias is generally low^43^.

We found that the GMV of TS patients decreased in bilateral IFG, which was thought to be responsible for the inability of TS patients to control their behavior^15^. The IFG has been suggested as the main brain region involved in response inhibition^44–48^, which occurs throughout its connection with the motor system^46,49^. Neuronal dysfunction of the IFG may lead to motor impulsivity, which is closely related to the core symptom of TS patients, i.e., involuntary tics^12,50–54^. In addition, OCD and ADHD patients often share tics and obsessive-like behaviors, which are characterized by repetitive, unconscious, involuntary and stereotyped movements^55,56^. Studies have reported decreased GMV in the left IFG of OCD patients^57–59^ and in the right IFG of ADHD patients^60^. Structural and functional MRI studies suggest that deficiency of the right inferior frontal lobe is the basis of impaired response inhibition^61,62^. Reduction of the GMV in the IFG in these comorbidities may also result in deficiency of response inhibition or failure to control behavior and may consequently lead to tics^15,63^. Moreover, the left IFG may constitute a common potential neurological correlation between TS and OCD/ADHD^15^.

This meta-analysis revealed increased GMV in the right striatum (including the putamen) of TS patients. The striatum is the primary input nucleus in the basal ganglia. Neural information from the sensory, motor, and marginal cortical inputs are selected by the striatum to perform neurological functions such as motor control, habit formation, and some social behaviors^64–67^. Much attention has been given to the dopaminergic system and the γ-amino butyric acid (GABA)-ergic system of the striatum in TS patients^68^. The dopaminergic system is dysfunctional in TS patients^27,69–71^, which has been attributed to the impaired putamen in Lerner’s study^72^. An early study has shown that TS is associated with increased striatal dopaminergic innervation^73^. Hyperactivity of the dopamine system enhances striatal activity such as habit formation, initiation, and execution^18,65^, and thus promotes the formation of tics habits through the reinforcement of the learning process^74^. It has also been suggested that changes in GMV in the striatum are the morphological evidence of dopaminergic hyperfunction^17^. The striatum is also a part of the neural circuit that produces and controls movements through GABA^54,71,75–77^, an important inhibitory neurotransmitter. Tics are often considered an involuntary movement that can be suppressed. In the striatum, a decrease in GABA_A_ receptor binding and a decrease in GABAergic inhibitory neurons^78^ result in dysfunction of the GABAergic system^75,79,80^ and may lead to tics and obsessive-like behaviors in TS patients^67,81^. Future studies with different methodologies are expected to clarify how neurotransmitter changes may lead to volumetric abnormalities. Furthermore, the putamen, which is thought to be involved in habit learning and motor control^82^, showed increased GMV. Several functional studies have demonstrated increased activity of the putamen^72,83^ and a positive correlation with the severity of tics^83^. The increased activity of the putamen is a reflection of the increased signal properties caused by the change in neuronal volume^17^.

The thalamus is involved in cognitive and motor motivational pathways^19,84^ and multisensory integration^13^. We found that the GMV increased in the thalamus, and the blob for the effect reported in the thalamus appeared to lie mainly in the pulvinar nucleus (Fig. 2), which was consistent with a previous study^85^. One hypothesis to explain this phenomenon is based on the theory of compensatory mechanisms^16,85,86^, which suggested that thalamic GMV increases as an adaptive change to attenuate and control tics because the extended network formed by the expanded thalamus can increase the executive control of motor circuits in TS patients^85^. Another possible explanation is the dysfunction of motor circuity in TS patients. Overactivity of the output pathway of the basal ganglia may remove the inhibition of thalamocortical projections and result in overactivity of the motor nuclei of the thalamus^85^. Overactivity over an extended period of time may eventually result in activity-dependent hypertrophy in the thalamus^85^.

We found increased GMV in the anterior cerebellum, including the vermis and the left hemispheric lobule, which may suggest that the cerebellum is involved in the pathogenesis of TS. The cerebellum plays a role in motor control and some cognitive functions^87^. Activation of the cerebellar hemispheres and vermis during tic release has been observed in a functional study^72^, suggesting that the cerebellum may play an important role in tics of TS. The relationship between structural and functional changes needs to be validated by more studies with larger sample size and a longitudinal design.

As mentioned above, GM volumetric abnormalities in the IFG, putamen, thalamus, and other parts were found, most of which belonged to the cortico-striato-thalamo-cortical (CSTC) network^53^ and they interacted with one another. Dysfunction of the CSTC network has been widely recognized by neuropathological studies as well as structural and functional neuroimaging research^68,88–90^. It is generally believed that the striatum (especially the putamen and caudate nuclei) can inhibit the basal ganglia output nucleus through increased striatal activity when receiving the excitatory input from the cerebral cortex. Next, the inhibition of the thalamus is released, and the cortex is stimulated to generate tics^89^. The cerebellum, as a node outside of the classical CSTC network, is involved in the so-called “basal ganglia-cerebellar-thalamo-cortical system”^88^. Previous studies have suggested that the cerebellum, similar to the basal ganglia, integrates inputs from the cerebral cortex such as the prefrontal cortex, which then generates output to the anterior motor cortex, the primary motor cortex, and even the same areas of the input cortex, through the thalamus^91^. It was further found that the subthalamic nucleus in the basal ganglia had projections to the cerebellar cortex and integrated functions of the basal ganglia and the cerebellum^91^. Basal ganglia-cerebellar-cortical interactions play an important role in the generation of tics^88^. These brain regions may serve as new targets for further study to develop effective treatments. Some studies have shown that the application of deep brain stimulation to the CSTC nodes or the connections between the nodes may help to alleviate tics^92–94^. The cerebellum outside the classical network can also be a target for intractable TS^95^.

In the meta-regression analysis, we did not find significant associations between clinical variables and GMV changes. The negative results may be due to differences in age, disease duration, medication status and comorbidities among the TS patients included in the study. For example, tics in adults do not fluctuate as much as in children and adolescents, and such differences may also influence the scoring of disease severity. However, the mean age and YGTSS were still of particular interest to us, and the prospective assessment of these variables will still be useful for future research.

### Limitations

Our research has several limitations. First, compared with other meta-analyses, our study is based on the published coordinates of the original study instead of the raw data, which may lead to a bias in the results. Differences in the original studies may have had some effects, such as the use of MNI templates in children, incomplete clinical information, and different scanner parameters for data acquisition and postprocessing. Collecting original data and making efforts to minimize the differences of data from different sources of examining facilities may help to control the bias. Second, since the number of included studies in our study did not meet the minimum requirement of subgroup analyses^42^, we were unable to test the effects of age (pediatric vs. adult) or sex. Finally, only ten original studies were included; therefore, the results of the meta-regression would be affected. We expect that studies in the future will better verify our conclusions.

## Conclusions

This meta-analysis confirmed GMV changes in the IFG, striatum, thalamus, and cerebellum in TS patients, most of which are key nodes of the CSTC network. These findings provide new insights into the possible treatment targets of TS patients. However, they need to be confirmed by more studies, and the mechanism of GMV changes as well as the relationship between GMV changes and clinical symptoms need to be further clarified.

## Supplementary information

supplimentary material
