# Supplementary material for: Gray matter abnormalities in Tourette Syndrome: a meta-analysis of voxel-based morphometry studies
Source: Transl Psychiatry. 2021 May 14;11:287. doi: 10.1038/s41398-021-01394-8 (PMC8121885; doi:10.1038/s41398-021-01394-8)
Supplement: Supplementary file 1 — supplimentary material [file 41398_2021_1394_MOESM1_ESM.docx]

**Gray matter abnormalities in Tourette Syndrome: a meta-analysis of voxel-based morphometry studies.**

Xinyue Wan^1#^, Simin Zhang^1#^, Weina Wang^2^, Xiaorui Su^1^, Jun Li^3^, Xibiao Yang^4^, Qiaoyue Tan^1^, Qiang Yue^4*^, Qiyong Gong^1,5,6*^

**SUPPLEMENTARY MATERIAL**

*

*


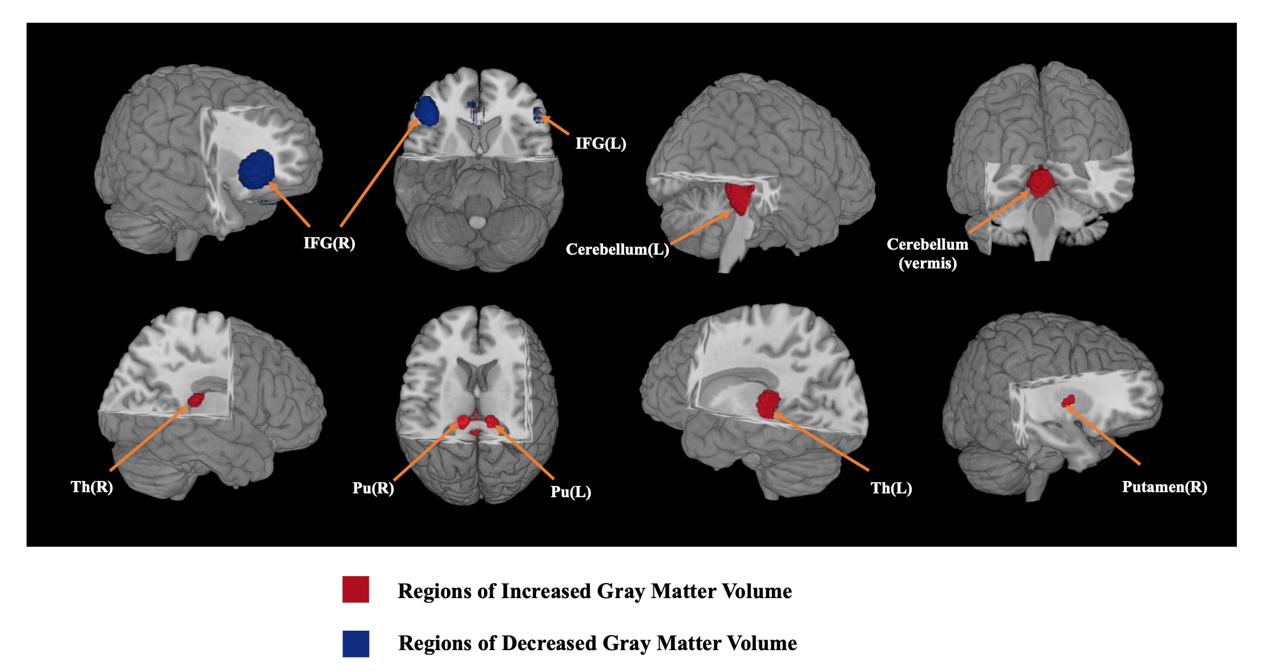


**Supplementary Fig. S1.** Regions showing gray matter volume alterations in TS patients (3D rendering)

***Abbreviation:*** TS, Tourette syndrome; L, Left; R, Right; IFG, Inferior frontal gyrus; Pu, the pulvinar nucleus; Th, thalamus.

**Supplementary table S1.** Preprocessing approaches employed in voxel-based morphometry analysis

| **Study** | **spatial normalization** | **bias field correction** | **segment** | **modulation** | **smooth** | **correction** |
| --- | --- | --- | --- | --- | --- | --- |
| Garraux et al., 2006 | Yes | NA | Yes | Yes (Jacobian determinants) | Yes | Total- GMV |
| Ludolph et al., 2006 | Yes | NA | Yes | Yes (Jacobian determinants) | Yes | Total-GMV |
| Muller-Vahl et al., 2009 | Yes (non-linear) | NA | Yes | Yes (Jacobian determinants) | Yes | The global mean voxel value of each tissue |
| Roessner et al., 2009 | Yes (non-linear) | yes | Yes | Yes | Yes | NA |
| Draganski et al., 2010 | Yes (DARTEL) | Yes | Yes | Yes (Jacobian determinants) | Yes | TIV |
| Wittfoth et al., 2012 | Yes (DARTEL ) | Yes | Yes | NA | Yes | NA |
| Liu et al., 2013 | Yes | NA | Yes | Yes | Yes | NA |
| Ganos et al., 2014 | Yes | NA | Yes | Yes (Jacobian determinants) | Yes | NA |
| Debes et al., 2014 | Yes (FLIRT + FNIRT) | NA | Yes | Yes (Jacobian determinants) | Yes | NA |
| Greene, et al.2017 | Yes | NA | Yes | Yes | Yes | Total-GMV |

***Abbreviations:*** DARTEL, Diffeomorphic Anatomical Registration Through Exponentiated Lie Algebra; FLIRT, FMRIB’s Linear image registration tool; FNIRT, FMRIB’s Non-linear image registration tool; FMRIB, Functional Magnetic Resonance Imaging of the Brain Software Library; NA, not available.

**Supplementary table S2.** Summary of results of articles included.

| **Study** | **Group for age** | **Regions (GMV increased** **in TS)** | **Regions (GMV decreased in TS)** |
| --- | --- | --- | --- |
| Liu et al., 2013 | Children | Bilateral precentral gyrus | Left superior temporal gyrus |
| Ludolph et al., 2006 | Adolescents | Bilateral ventral putamen | Bilateral hippocampal area |
| Greene, et al.2017 | Adolescents | Posterior thalamus, hypothalamus and midbrain | - |
| Roessner et al., 2009 | Adolescents | - | - |
| Debes et al., 2014 | Adolescents | Left putamen | - |
| Draganski et al., 2010 | Adults | Bilateral putamen | Orbitofrontal, anterior cingulate and bilateral ventrolateral prefrontal cortices, bilateral operculum. |
| Wittfoth et al., 2012 | Adults | - | Left inferior frontal gyrus |
| Garraux et al., 2006 | Adults | Left midbrain | - |
| Ganos et al., 2014 | Adults | - | Right inferior frontal gyrus, left frontal pole |
| Muller-Vahl et al., 2009 | Adults | - | prefrontal areas, the anterior cingulate gyrus, sensorimotor areas, left caudate nucleus and left postcentral gyrus |

***Abbreviations:*** GMV, gray matter volume; TS, Tourette syndrome.

**Supplementary table S3.** The original coordinates with respective effect size of included studies

| Study | MNI Coordinates | | | Peak t |  |  |  |  |  |
| --- | --- | --- | --- | --- | --- | --- | --- | --- | --- |
|  | x | y | z |  |  |  |  |  |  |
| Garraux et al., 2006 | -7 | -29 | -14 | 4.258 |  |  |  |  |  |
|  | -2 | -13 | -11 | 3.112 |  |  |  |  |  |
|  | -7 | -17 | -4 | 3.051 |  |  |  |  |  |
| Ludolph et al., 2006 | 25 | 13 | -5 | p |  |  |  |  |  |
|  | -20 | 17 | -6 | p |  |  |  |  |  |
|  | 27 | -19 | -17 | n |  |  |  |  |  |
|  | -20 | -19 | -21 | n |  |  |  |  |  |
| Muller-Vahl et al., 2009 | -55 | -8 | 20 | -5.04 |  |  |  |  |  |
|  | 38 | -20 | 47 | -4.54 |  |  |  |  |  |
|  | 9 | 22 | 31 | -4.01 |  |  |  |  |  |
|  | -15 | 14 | 13 | -4.00 |  |  |  |  |  |
|  | -10 | 11 | 3 | -3.54 |  |  |  |  |  |
|  | -36 | 12 | 56 | ,-4.00 |  |  |  |  |  |
|  | -45 | -18 | 36 | -3.96 |  |  |  |  |  |
|  | -52 | -15 | 40 | -3.52 |  |  |  |  |  |
|  | 44 | 6 | 57 | -3.96 |  |  |  |  |  |
|  | 59 | -13 | 38 | -3.95 |  |  |  |  |  |
|  | 45 | -19 | 38 | -3.95 |  |  |  |  |  |
|  | 41 | -26 | 58 | -3.68 |  |  |  |  |  |
|  | 10 | 29 | 48 | -3.62 |  |  |  |  |  |
|  | -9 | 12 | 39 | -3.61 |  |  |  |  |  |
| Roessner et al., 2009 | - | - | - | - |  |  |  |  |  |
| Draganski et al., 2010 | -53 | 35 | -6 | -4.8 |  |  |  |  |  |
|  | 45 | 38 | -12 | -4.9 |  |  |  |  |  |
|  | 0 | 33 | -26 | -5.3 |  |  |  |  |  |
|  | -39 | -3 | 7 | -4.4 |  |  |  |  |  |
|  | 58 | -13 | 16 | -3.9 |  |  |  |  |  |
|  | 7 | 44 | 1 | -3.7 |  |  |  |  |  |
|  | -27 | -10 | 3 | 3.3 |  |  |  |  |  |
|  | 27 | -3 | 9 | 4.5 |  |  |  |  |  |
| Wittfoth et al., 2012 | 36 | 38 | 3 | -3.44 |  |  |  |  |  |
| Liu et al., 2013 | -58 | -36 | 14 | -5.29 |  |  |  |  |  |
|  | -12 | -28 | 72 | 3.87 |  |  |  |  |  |
|  | 18 | -24 | 68 | 3.44 |  |  |  |  |  |
| Ganos et al., 2014 | 32 | 25 | -14 | n |  |  |  |  |  |
|  | -20 | 60 | 4 | n |  |  |  |  |  |
| Debes et al., 2014 | -26 | -6 | 14 | p |  |  |  |  |  |
|  | -13.5 | -30 | 9 | 4.62 |  |  |  |  |  |
|  | -15 | -28.5 | -4.5 | 4.26 |  |  |  |  |  |
|  | 0 | -33 | -4.5 | 3.75 |  |  |  |  |  |
|  | 0 | -34.5 | -13.5 | 3.46 |  |  |  |  |  |
| Greene, et al.2017 | 9 | -3 | -16.5 | 4.06 |  |  |  |  |  |
|  | -1.5 | -6 | -7.5 | 3.93 |  |  |  |  |  |
|  | 0 | -15 | -10.5 | 3.76 |  |  |  |  |  |
|  | 16.5 | -28.5 | -4.5 | 4.13 |  |  |  |  |  |
|  | 12 | -30 | 12 | 3.9 |  |  | | |  |

***Abbreviations:*** p, positive peaks; n, negative peaks.
